# Diagnostic and predictive value of structural and high-order functional brain topology in major depressive disorder

**DOI:** 10.3389/fpsyt.2026.1964608

**Published:** 2026-10-20

**Authors:** Xiaoyan Wang, Xiaofeng Wu, Mingqi Shi, Kaili Che, Xiaojun Shen, Tongpeng Chu, Ning Mao, Peiyuan Wang

**Affiliations:** 1 Department of Radiology, Yantai Affiliated Hospital of Shandong Medical and Pharmaceutical University, Yantai, Shandong, China; 2 School of Medical Imaging, Shandong Medical and Pharmaceutical University, Yantai, Shandong, China; 3 Department of Radiology, Yantai Yuhuangding Hospital, Qingdao University, Yantai, Shandong, China; 4 Shandong Provincial Key Medical and Health Laboratory of Intelligent Diagnosis and Treatment for Women’s Diseases (Yantai Yuhuangding Hospital), Yantai, China; 5 Big Data and Artificial Intelligence Laboratory, Yantai Yuhuangding Hospital, Qingdao University, Yantai, Shandong, China; 6 Shandong Provincial Key Laboratory of Neuroimmune Interaction and Regulation, Yantai, Shandong, China

**Keywords:** brain network organization, higher-order interactions, machine learning, major depressive disorder, multimodal neuroimaging

## Abstract

**Background:**

Conventional neuroimaging analyses for major depressive disorder (MDD) predominantly construct brain networks based solely on pairwise regional functional couplings. Accumulating microscale neuroscientific evidence highlights the essential role of higher-order interactions (HOIs, interactions among three or more brain nodes) in capturing the intricate spatiotemporal dynamics of the human brain. This study aims to integrate machine learning techniques with neuroimaging topological features to systematically investigate the impact of non-pairwise interaction patterns in the brains of MDD patients on system dynamics, and to develop diagnostic and predictive models for MDD clinical manifestations.

**Methods:**

Firstly, diffusion tensor imaging (DTI) and resting-state functional magnetic resonance imaging (rs-fMRI) data were acquired from 154 MDD patients with first-episode, drug-naïve and 153 age and gender matched healthy controls (HCs). Graph-theoretical analysis was applied to DTI-derived tractography data to quantify structural topology metrics, whereas persistent homology was adopted to extract higher-order functional topological signatures from rs-fMRI time series. Second, feature-level fusion was performed, and then a classification model was constructed using support vector machine (SVM) for MDD diagnosis. A repeated nested validation strategy with 50 repeated stratified 70/30 hold-out splits was adopted. Classification performance was evaluated using the area under the receiver operating characteristic curve (AUC). Subsequently, a prediction model was built based on the selected fused features using ridge regression, and the prediction performance was evaluated based on the correlation between the predicted values and the actual scale scores.

**Results:**

Compared with HC, MDD patients exhibited significant alterations in structural and functional topological metrics within key brain networks, including DMN, VN and FPN. The classification performance of the SVM model integrating multimodal neuroimaging features achieved excellent classification performance, with an AUC of 88.2%. Additionally, multimodal imaging features demonstrated robust predictive capacity for suicide risk level in MDD patients (r=0.604).

**Conclusion:**

By integrating structural and higher-order functional topological features, we are able to effectively diagnose and predict MDD, highlighting the crucial role of higher-order interactions in the pathological mechanisms of brain networks in depression.

## Introduction

Major depressive disorder (MDD) is one of the most common and debilitating psychiatric illness that affects over 320 million people around the world and imposing a substantial global burden (1, 2). It is characterized by persistent depressed mood, anhedonia and worthlessness, collectively accounting for significant negative effects on an individual’s social interactions, personal relationships, and professional functioning (3, 4). Despite significant advances in neuroscience and antidepressant therapy over the past few decades, the neurobiological mechanisms underlying MDD remains elusive (5).

Modern neuroimaging studies have demonstrated extensive structural and functional impairments in MDD patients (6–8), characterized by alterations in white matter integrity and functional connectivity across large-scale brain networks, many of which are associated with symptom severity (9, 10). The structural connectivity of the brain mainly refers to the anatomical connections formed between brain regions through white matter fiber tracts, reflecting the physical basis for information transmission within the brain (11, 12). Diffusion tensor imaging (DTI) is an advanced magnetic resonance imaging (MRI) technique used to non-invasively measure the direction and extent of water molecule diffusion in brain tissue (13–15). A previous follow-up study on white matter integrity in MDD patients demonstrated reduced fiber integrity in large clusters including the corpus callosum and corona radiata compared with healthy controls (HCs), negatively correlated with cognitive performance (16). DTI-based structural topological analysis combines white matter fiber tracking techniques with graph theory to characterize the features of the brain’s structural connectivity network. Previous studies have found that patients with MDD exhibit impaired connectivity within the prefrontal-limbic system and the default mode network (DMN), accompanied by reduced network efficiency and abnormal small-world properties (17, 18).

It is worth noting that structural connectivity provides the anatomical basis for brain function, whereas functional networks reflect the synergistic interactions among different brain regions. Functional connectivity (FC) reflects the temporal correlation of neural activity between different brain regions and is regarded as the most widely applied brain network model (19, 20). Resting-state functional magnetic resonance imaging (rs-fMRI) characterizes patterns of functional connectivity by measuring the temporal correlations in bold oxygen level-dependent (BOLD) signals between brain regions. Compared with HCs, MDD patients show significantly reduced FC between the default mode network (DMN) and the dorsal attention network (DAN), and alterations in DMN-DAN connectivity are closely associated with depressive symptoms (21, 22). These alterations may have potential value for discriminating individuals with MDD from HCs.

Despite the widespread adoption of FC, traditional analytical methods typically concentrate on pairwise interaction networks among brain regions. However, brain function relies on coordinated interactions among multiple brain regions, and increasing evidence suggests that higher-order interactions (HOIs) involving three or more nodes provide a more comprehensive characterization of brain dynamic (23–25). Some studies have pointed out that higher-order methods can enhance the brain’s ability for dynamically decoding among different tasks and significantly strengthen the correlation between brain activity and behavior (26). By considering the higher-order (group) interactions in more refined models, such as hypergraphs and simplicial complexes, profound and qualitative changes can be triggered in the dynamics of the brain system (27–31). In addition, by combining network analysis and topological methods, this approach reconstructs higher-order interactions among node-based multivariate time series (MTS) (32).

However, although the techniques employed in animal studies can record high-order phenomena at the microscale level, these techniques have not yet been applied in human research (33, 34). Therefore, researchers have to rely on indirect methods to infer high-order interactions from the time series of nodes activity through statistical analysis. Recent information-theoretic studies provides evidence for the existence of high-order interactions in the brain, which can be reconstructed through functional magnetic resonance imaging (fMRI) and significantly explain the complex dynamics of brain function (32, 35, 36). Moreover, early instances of inferring temporal HOI statistics have been successfully used as features in machine learning classifiers, providing higher accuracy compared to methods based on pairwise descriptions (37).

Despite recent advances in neuroimaging research have provided crucial insights into the neurobiological mechanisms of MDD, several challenges remain. First, most studies rely on traditional functional connectivity or single-modal imaging, limiting the characterization of complex brain network organization. Second, higher-order functional network analysis can identify complex interactions beyond pairwise connectivity, yet its complementary relationship with structural remains largely unexplored. Given the lack of objective biomarkers for MDD, integrating multimodal brain network information to develop robust classification and prediction models remains an important research goal.

This study aimed to combine the traditional structural networks and the functional networks of high-order interactions, and to evaluate their utility in MDD classification and symptom prediction using multimodal fusion and machine learning approaches. **Figure 1** illustrates the workflow.

**Figure 1:**
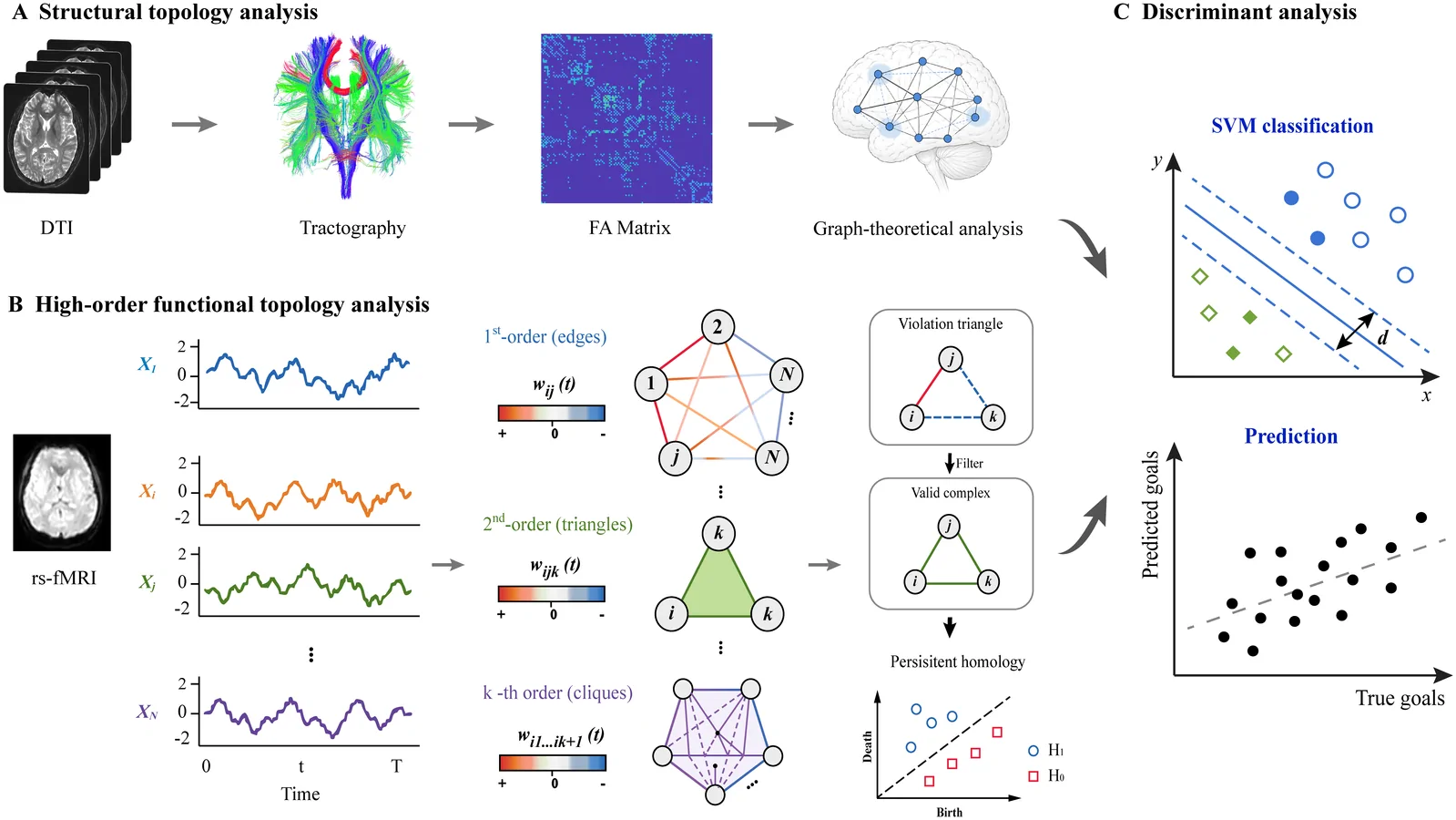
The analytical workflow of this study. **(A)** Topological analysis of brain structural networks from DTI data using fiber-tracing technology and graph theory. **(B)** Higher-order topological analysis of fMRI time series: after z-score normalization, pairwise and triple-wise co-fluctuations are computed to build a weighted simplicial complex. A filtration process with simplex-closure constraint is applied (k=2); violating triangles are recorded for hyper-coherence calculation. Persistent homology is then performed to extract 1-dimensional cycles and compute the persistence scaffold. **(C)** Discriminant analysis: Using machine learning model to diagnose and predict MDD patients. DTI, diffusion tensor imaging; rs-fMRI, resting-state functional magnetic resonance imaging; FA, fiber tractography; SVM, support vector machine.

## Materials and methods

### Participants

This study was approved by the Medical Research Ethics Committee of Yantai Yuhuangding hospital and informed consent was obtained from all participants. All procedures were performed in accordance with ethical guidelines and regulations. A total of 177 patients with depression were recruited from Yantai Yuhuangding Hospital, and 167 healthy controls matched in age and gender were recruited from the local community. During quality control and analysis, participants with poor MRI quality or incomplete clinical scale data were excluded, leaving 154 MDD patients and 153 HCs for demographic and clinical analyses. All patients with depression were diagnosed by qualified geriatric psychiatrists based on the criteria for major depressive episode in the fifth edition of the Diagnostic and Statistical Manual of Mental Disorders (DSM-V) (38). The Hamilton Depression Rating Scale (HAMD), Montreal Cognitive Assessment (MoCA), and Nurse Global Assessment of Suicide Risk (NGASR) scores of all participants were collected during the recruitment stage for subsequent analysis.

The inclusion criteria for patients with MDD are as follows (1): age range: 18–60 years old; (2) handedness and education: right-handed patients with a minimum education level of 6 years; (3) depression severity: HAMD-24 score ≥ 20. The exclusion criteria include: (1) other types of mental illnesses apart from MDD; (2) history of alcohol or drug abuse; (3) concurrent major diseases of other types; (4) contraindications examination for MRI; (5) women who are pregnant or breastfeeding. The HCs were matched with the MDD patients in terms of age, gender and education level. The inclusion criteria for the control group include (1) the score of HAMD ≤ 8; (2) right-handedness; (3) no history of any mental illness. The exclusion criteria applied to the HCs are consistent with those for the patients with MDD.

### Clinical measurements

The HAMD scale is a classic tool for clinical assessment of depressive states. It covers 7 core dimensions and consists of 24 questions, each with 5 response options, scored from 0 to 5 points. The higher the total score, the more severe the depression. The reliability and validity of this scale have been widely verified in China and it has high sensitivity to depressive symptoms in neurology patients, making it a commonly used tool in neuroimaging studies to correlate with depressive phenotypes. Cognitive assessment for all participants was conducted using the Chinese version of the MoCA (39). The MoCA is a widely used screening tool for mild cognitive impairment (MCI) internationally, covering multiple cognitive domains. Its assessment includes visual-spatial skills, executive function, naming ability, memory, attention span, language ability, abstract reasoning, delayed recall, and orientation. The higher the score, the better the cognitive function. A total score of ≥26 is considered normal cognitive function and can effectively identify early cognitive impairment in neurology patients. Suicide is a multi-faceted and complex phenomenon, which makes the assessment of suicide risk and its related factors extremely challenging. The NGASR, as a highly trusted suicide risk assessment scale, focuses on multi-dimensional evaluation of patients’ suicide risk (40).

### MRI acquisition and preprocessing

In this study, researchers acquired rs-fMRI and diffusion tensor imaging (DTI) data by using a 3.0 T Ingenia CX MR scanner (Philips Healthcare, the Netherlands). In addition, T1-weighted images were acquired for subsequent image registration, along with T2-weighted and fluid-attenuated inversion recovery (FLAIR) sequences to rule out anatomical lesions. The specific sequence parameters are shown in **Table 1**.

**Table 1:** MRI sequence parameters.

Sequence	TR (ms)	TE (ms)	FA (°)	FOV (mm^3^)	voxel size (mm^3^)	Slice number	Slice thickness (mm)	Matrix size
rs-fMRI	2000	30	90	224×224	3.5×3.5×4	36	4	64×61
DTI	7931	73	90	222×222	2.0×2.0×2.0	70	2	112×108
3D-T1	8.1	3.7	8	224×224	1.0×1.0×1.0	172	1	224×224

rs-fMRI, resting-state functional MRI; DTI, Diffusion Tensor Imaging; TR, repetition time; TE, echo time; FA, flip angle; FOV, field of view.

The preprocessing of DTI data were conducted using the pipeline for analysis of brain diffusion images (PANDA, http://www.nitrc.org/projects/panda/) (41). Firstly, the DICOM format images were converted to NIFTI format and the skull was removed to extract the brain tissue. Then, head motion correction and eddy current correction were performed to the DTI data. Next, the individual T1-weighted sequence images were registered with the DTI images and the registered images were normalized to the Montreal Neurological Institute (MNI) space. Finally, smoothing was applied to alleviate the registration errors and enhance the gaussianity of the data.

The functional imaging data were pre-processed using the resting-state fMRI data processing toolbox (DPARSF v5.2, http://rfmri.org/dparsf) (42). Firstly, the data format was converted from DICOM to NIFTI. Then, the first 10 time points were discarded to eliminate the initial unstable signals caused by factors such as equipment startup and subjects’ adaptation to the scanning environment. Subsequently, temporal slice correction and head motion correction were performed, and the images were normalized to the Montreal Neurological Institute space (MNI), with each voxel resampled to 3×3×3 mm³, enabling the comparison and analysis of images from different subjects within the same spatial coordinate system. Finally, a 6mm full width at half maximum Gaussian smoothing kernel was applied to the images for smoothing. Additionally, a band-pass filter of 0.01-0.10 Hz was implemented to remove high-frequency noise and low-frequency drift while retaining signals related to neural activity.

### Structural topology analysis

Diffusion data preprocessing and network construction were conducted using PANDA. Deterministic fiber tracking was implemented by using the fiber assignment by continuous tracking (FACT) algorithm with Diffusion Toolkit (http://trackvis.org/dtk/). Fiber tracking was terminated when the crossing angle between two consecutive movement directions exceeded 45° or when the fractional anisotropy (FA) was outside the threshold range of 0.2-1. The Automated Anatomical Labeling (AAL) atlas was employed for whole-brain tractography reconstruction, parcellating the brain network into 116 nodes, comprising 90 cerebral regions and 26 cerebellar regions. Nodes correspond to regions of interest from the AAL atlas, while edges are defined as FA values of reconstructed white matter tracts connecting these regions. Therefore, we acquired the 116×116 structural connectivity matrices representing the anatomical networks for each subject. We preprocessed the FA matrix by removing the diagonal elements, applying symmetrization, and eliminating outliers, thereby transforming the original matrix into a weighted network suitable for subsequent analysis. Subsequently, the acquired structural brain networks were analyzed using the GRETNA software (http://www.nitrc.org/projects/gretna/) package for graph-theoretical analysis.

### Higher-order topological analysis of multivariate time series

To quantify the high-order topological properties of brain functional networks, we employed a multivariate time series analysis method based on persistent simplicial homology (32). The same AAL 116 atlas was used for both structural and functional network construction. Firstly, for each individual, we extracted the time series $x_{i}$ for each brain region and performed z-score normalization, such that $z_{i(t)}=\frac{x_{i(t)}-\mu_{i}}{\sigma_{i}}$, where $x_{i(t)}$ is the original signal of brain region the mean and standard deviation of region i’s time series, respectively. As in the reference (32), in this work we also consider only co-fluctuations of dimension k = 2. Therefore, we calculated the product time series for all brain region pairs (edges) and brain region triplets (triangles), which represent second-order and third-order functional interactions, respectively (Equations 1, 2).

(1) $$\text{second}-\text{order}:\;w_{ij(t)}=\frac{z_{i(t)}\cdot z_{j(t)}-u_{ij}}{\sigma_{ij}}$$

(2) $$\text{third}-\text{order}:\;w_{ijk(t)}=\frac{z_{i(t)}\cdot z_{j(t)}\cdot z_{k(t)}-u_{ijk}}{\sigma_{ijk}}$$

To ensure the physical interpretability of the interaction weights, we incorporated a coherence correction mechanism. This mechanism modifies the weights through an analysis of the sign consistency of the original signals, thereby ensuring that a fully coherent state (in which all signals exhibit the same sign) yields a positive weight, while an incoherent state corresponds to a negative weight (Equation 3).

(3) $$\text{coherence}(v)={\left(-1\right)}^{\text{sign}(n-|\sum_{k=1}^{n}\text{sign}(v_{k})|)}$$

Here, v denotes the raw signal vector of brain regions, and sign(·) refers to the sign function. This consistency function yields 1 for a fully coherent state and -1 for one that is not fully coherent. According to the consistency state, raw interaction weights are corrected as follows (Equation 4):

(4) $$w_{\text{corrected}}=\{\begin{array}{cc} \left|w\right| & \text{if coherence}=1\\ -|w| & \text{if coherence}=-1 \end{array}$$

In our methodology, the primary focus centers on the contribution of internal consistency of the multivariate time series, specifically the fully coherent state.

For each time point t, we establish a time-evolving weighted simplicial complex $K_{t}$ in accordance with a functional connectivity strength threshold. Formally, a (d−1)-dimensional simplex σ is defined as consisting of d vertices, i.e., $\sigma =[p_{0},p_{1},\ldots ,p_{d-1}]$. A collection of simplices constitutes a simplicial complex K if, for every simplex σ contained within K, all its possible sub-faces (defined as subsets of σ) are likewise included (43). A weighted simplex refers to a simplex to which a value (referred to as its weight) has been assigned. Following the approach outlined in the cited reference, this work also focuses exclusively on co-fluctuation sequences for triadic interactions; so that triangles serve as the only higher-order structures within the weighted simplicial.

To examine the structure of the weighted simplicial complex $K_{t}$ across multiple scales, we rely on persistent homology, a cutting-edge technique in computational topology that has been widely applied to the analysis of high-dimensional datasets (44, 45) and diverse applications (46–48). The core idea lies in the construction of a nested sequence of simplicial complexes, which progressively refines the approximation of the original weighted complex with enhanced precision. Via a filtration process conducted in the order of descending weights, all triangles that don’t satisfy the simplicial closure condition are identified. These triangles are regarded as violations and added to a violation list together with their respective weights. Subsequently, the remaining weights that meet the condition are utilized to construct the simplicial complex.

We compute the hyper-coherence index, defined as the proportion of violating triangles relative to all potential coherent triangles.

Persistent homology investigates how topological structures evolve throughout the filtration process and offers a natural and robust metric for topological features that emerge across various scales. Specifically, these topological changes are monitored by examining each k-dimensional cycle within the homology group $H_{k}$. In the present study, we concentrate on 1-dimensional holes (i.e., loops) in the homology group $H_{1}$. At each filtration step, a generator g uniquely characterizes a 1-dimensional cycle via its constituent elements. The significance of a 1-dimensional cycle g is encoded by its birth timestamp $b_{g}$ and death timestamp $d_{g}$, which are documented throughout the filtration process (31). By combining these two timestamps, the persistence $\pi_{g}=d_{g}-b_{g}$ of the loop is defined, thereby quantifying its significance in terms of its lifespan.

The results of the persistent homology group $H_{1}$ are typically visualized as points in a two-dimensional persistence diagram. In this diagram, each point ( $b_{g}$, $d_{g}$) represents a one-dimensional hole g that appears throughout the filtration process. Consequently, the diagram offers a concise summary that depicts the lifespan of each 1D cycle during filtration and can act as a proxy for the “complexity” of the underlying space. Accordingly, we define the hyper-complexity index as the Wasserstein distance between the persistence diagram of $H_{1}$ and an empty persistence diagram, which corresponds to a space with trivial $H_{1}$ homology (49). This provides us a metric to quantify the topological complexity of both coherent and incoherent co-fluctuation landscapes.

To better characterize the topological features exhibited in the persistence diagram, we utilize the persistent homology scaffold proposed in the cited literature (31). This scaffold is a weighted network that incorporates all cyclic paths corresponding to generators $g_{i}$, where weights are determined by the persistence values of their associated homology classes. Specifically, if an edge e is part of multiple 1-dimensional cycles $g_{0},g_{1},\ldots ,g_{s}$, its persistent weight is defined as the cumulative sum of the persistence values $p(g_{i})$ of these cycles, as defined in Equation 5:

(5) $$w_{e}^{p}=\sum_{g_{i}\in H_{1}}p(g_{i})\cdot 1_{\text{e}\in {\text{rep(g}}_{\text{i}})}$$

Here, $1_{\text{e}\in {\text{rep(g}}_{\text{i}})}$ is an indicator function that takes the value of 1 if edge e is present in the representative cycle of the homology class $g_{i}$, and 0 otherwise. The information furnished by the persistence scaffold allows us to dissect the roles of different connections in the system’s topological stability. For a connection e, a higher persistent weight $w_{e}^{p}$ indicates its involvement in topological features that remain stable across a wide parameter range, which reflects the robustness of the network topology (31). Connections with high persistent weights constitute the topological backbone of the system. They maintain stable loop structures within both coherent and incoherent co-fluctuation landscapes, and are thus critical to the overall topological integrity of the system.

Previous studies have demonstrated that both the violating triangles and homological scaffolds can effectively decode information during tasks (26). Accordingly, our study focuses on the local organization of the brain functional architecture. As local indictors, we employ the identities and weights of violating triangles and the homological scaffolds, both derived during step of the topological data analysis pipeline. The identities and weights of violating triangles capture higher-order coherent co-fluctuations that cannot be fully characterized by pairwise connections (i.e., edges). The homological scaffold (31) quantifies the functional relevance of individual edges in supporting mesoscopic topological features, such as 1-dimensional loops, within the higher-order co-fluctuation landscape. Thus, it highlights specific connections that play structurally pivotal roles in shaping large-scale patterns of brain activity.

### Statistical analysis

To examine the statistical difference in demographic characteristics between MDD patients and healthy controls. We conducted all statistical analyses using SPSS (version 27.0; IBM SPSS Statistics for Window). For continuous numerical data meeting normality assumptions (e.g., age, years of education, HAMD scores, MoCA scores, NGASR scores), we employed an independent samples t-test. Categorical data (e.g., gender) were analyzed using chi-square (χ2) tests.

Two-sample independent t-tests, adjusted for age and sex as nuisance covariates, were implemented to detect intergroup topological disparities. FDR correction was applied to account for multiple statistical testing and minimize false-positive findings, with the corrected significance threshold set at p < 0.05.

### Classification model

To integrate structural and functional information, a feature-level fusion strategy was used to construct multimodal features. A repeated nested validation strategy was adopted, consisting of 50 repeated stratified 70/30 hold-out splits. In each repetition, 70% of the samples were used as the outer training set and the remaining 30% were retained as an independent outer testing set. All data-dependent procedures were performed exclusively within the outer training set. Specifically, age, sex, and years of education were treated as nuisance covariates and adjusted using parameters estimated from the corresponding training data only. Z-score normalization was then performed using the training-set mean and standard deviation, which were subsequently applied to the corresponding validation or testing data. Within each outer training set, 10-fold inner cross-validation was used for feature selection and model development. Features were first screened using two-sample t-tests (p < 0.05) and further reduced using least absolute shrinkage and selection operator (LASSO) regression with 10-fold cross-validation. The selected features were then used to train a radial basis function support vector machine (SVM) classifier. The kernel scale parameters were automatically optimized using Bayesian optimization within the inner cross-validation procedure. The optimized hyperparameters for each model are reported in **Table 2**. Posterior probability estimation using Platt scaling was performed using training-derived out-of-fold predictions, and the optimal classification threshold was determined within the outer training set based on the Youden index (sensitivity + specificity − 1). The resulting model, calibration parameters, and classification threshold were then applied to the independent outer testing set. Classification performance was evaluated on the held-out outer testing set in each repetition using the area under the curve (AUC), accuracy, sensitivity, specificity, and F1-score. Performance across the 50 repetitions was summarized using empirical 95% intervals. Additionally, we constructed unimodal models based solely on structural features, solely on functional features, and a multimodal model that fused both types of features. We compared the performance of different classifiers on the independent outer test set to report their respective predictive efficacy. A conventional pairwise FC-based model was additionally constructed and compared with the HOF-based model.

**Table 2:** Results of optimizing the kernel parameters of RBF-SVM.

SVM model	KernelScale	BoxConstraint (C)	Gamma
Structural (DC)	8.566	37.671	0.00682
Functional (FC)	3.014	0.523	0.05510
Functional (HOF)	14.172	5.846	0.00250
Multimodal (DC+HOF)	10.604	2.470	0.00445

DeLong tests were used to compare ROC curves based on predicted probabilities obtained from the same held-out test subjects within each repetition. Statistical significance was defined as p<0.05.

### Predict MDD suicide risk

We developed a neuroimaging-based prediction model for NGASR (40). To reduce feature dimensionality and enhance model generalizability, we employed a filter-based feature selection strategy. Within each outer training set, neuroimaging features and clinical scores were standardized using the corresponding training-set means and standard deviations. Pearson correlation coefficients were then computed between each neuroimaging feature and clinical score to quantify their linear association strength. Features with an absolute correlation coefficient exceeding the predefined threshold were retained for predictive modeling.

For predictive modeling, we adopted Ridge Regression, a linear regression method incorporating an L2-norm regularization term, which effectively constrains model complexity and mitigates overfitting. Model evaluation was performed using nested 10-flod cross-validation. The outer 10-fold cross-validation within each outer-of-sample predictions, while an inner 10-fold cross-validation within each outer training set was used to determine the optimal regularization parameter (λ). Specifically, λ was selected from a logarithmically spaced grid ranging from 10^-4^ to 10^4^ by minimizing the mean squared prediction error across the inner folds. The model was then refitted on the complete outer training set using the selected λ and applied to the held-out test set. The primary metric for evaluating predictive accuracy was the Pearson correlation coefficient (r) and coefficient of determination (R^2^) between the predicted and actual clinical scores.

## Results

### Demographics and clinical characteristics

The demographic and clinical characteristics of MDD patients and healthy controls are summarized in **Table 3**. No significant group differences were observed in sex (χ^2^ = 0.823, P = 0.364), years of education (t = 1.241, P = 0.216) or age (t = 0.489, P = 0.625) between MDD patients and healthy controls. Compared with HCs, the MDD group exhibited significantly higher scores on the HAMD and the NGASR, and significantly lower scores on the MoCA.

**Table 3:** Demographic and clinical characteristics.

Characteristics	MDD	HCs	P-value
Age	32.16 ± 11.83	32.82 ± 11.62	0.625
Education	14.8 ± 3.07	15.33 ± 4.13	0.216
Sex(male/female)	43/111	50/103	0.364
HAMD	28.22 ± 10.35	2.58 ± 2.99	<0.001
MoCA	21.44 ± 2.57	27.92 ± 1.64	<0.001
NGASR	8.17 ± 2.93	NA	

MDD, Major Depressive Disorder; HC, healthy controls; HAMD-24, 24-item Hamilton depression scale; MoCA, Montreal Cognitive Assessment Scale; NGASR, Nurses Global Assessment of Suicide Risk; NA, not available.

### Network topological analysis

#### Structural topological differences

We employed traditional structural network topology methods for DTI data analysis. Compared with healthy controls, patients with major depressive disorder exhibited significant alterations in structural topological properties across multiple large-scale brain networks. Reduced degree centrality (DC) was predominantly observed in regions belonging to the DMN and the visual network (VN), whereas increased metrics were mainly detected in frontoparietal network (FPN) and salience/ventral attention network (SN/VAN) regions. Significant alterations were also observed in the bilateral Heschl’s gyrus, subcortical structures, limbic regions, and cerebellar regions (**Figure 2**).

**Figure 2:**
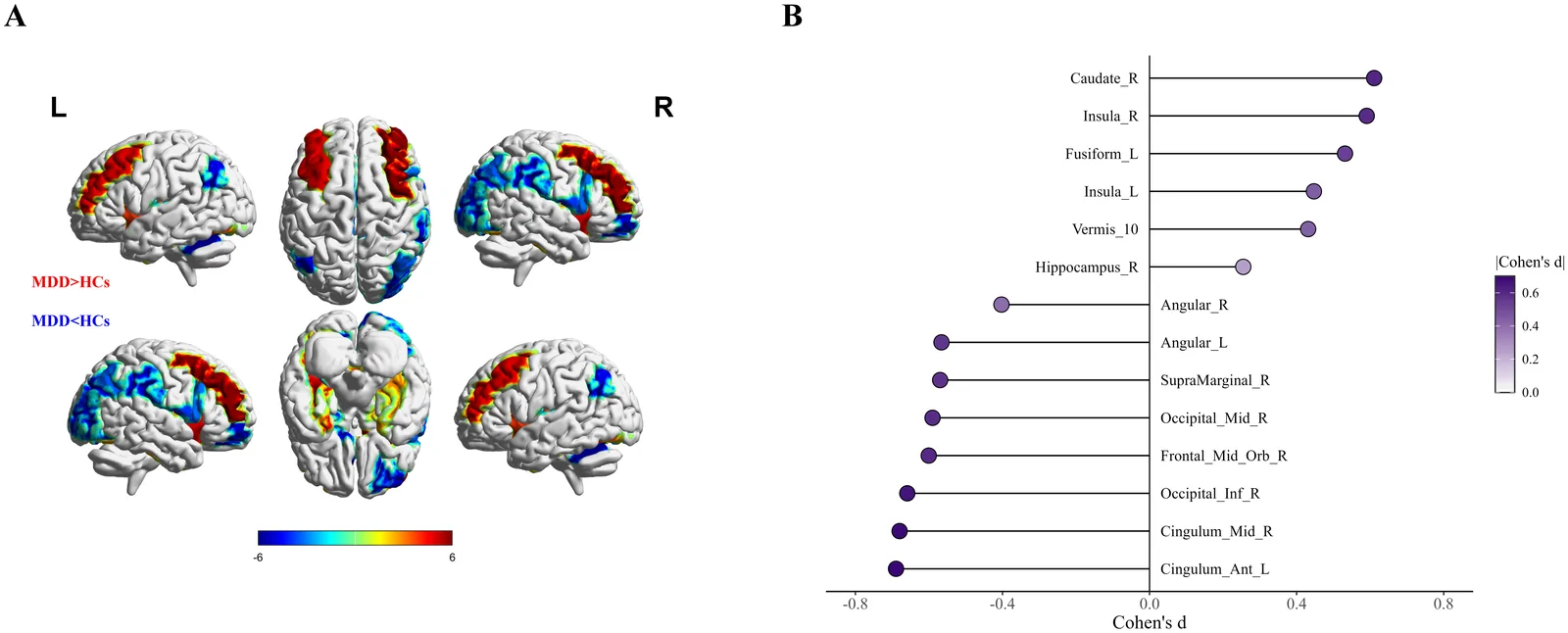
Abnormal degree centrality in MDD group. **(A)** Red/blue colors indicate increased/decreased DC in patients; color intensity represents t-value magnitude. **(B)** Effect sizes (Cohens’d) of DC alterations in significant brain regions. MDD, major depressive disorders; HCs, healthy controls; R, right; L, left.

#### Higher-order functional topological differences

Compared with HCs, individuals with depression exhibited significantly increased violating triangles across widespread brain regions. These alterations involved multiple large-scale brain networks, including the DMN, SN/VAN, FPN, VN, SMN, limbic, subcortical, and cerebellar systems. Particularly prominent increases were observed in DMN-related regions, such as the medial prefrontal cortex, cingulate cortex, hippocampal formation, and precuneus, as well as in the insula, frontal cortex, basal ganglia, thalamus, and cerebellum (**Figure 3**).

**Figure 3:**
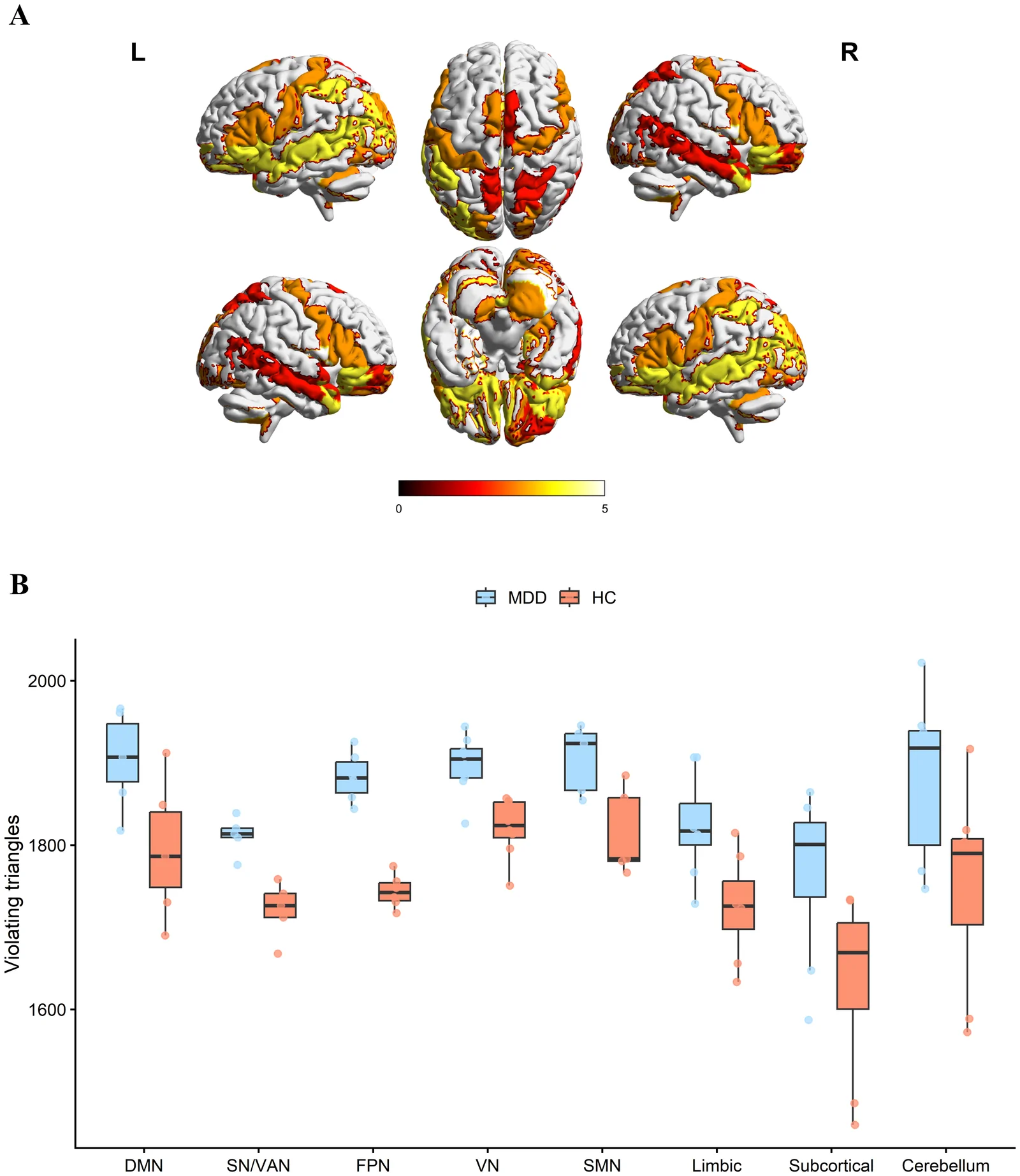
Increased violating triangles in MDD. **(A)** Brain regions with significant alterations. **(B)** Distribution across functional networks. MDD, major depressive disorders; HC, healthy control; R, right; L, left.

Two distinct patterns of abnormal homologous scaffolds were observed in the brain networks of the depression patients relative to healthy controls. Increased scaffold connectivity was predominantly observed among frontal, limbic and DMN-related regions, as well as among cortical, subcortical and cerebellar regions (**Figure 4A**). In contrast, decreased connectivity was primarily distributed within parietal–cerebellar, sensorimotor–parietal, thalamic, and frontostriatal pathways (**Figure 4B**). The specific connections involved in the two types of homologous scaffolds are detailed in **Table 4**.

**Figure 4:**
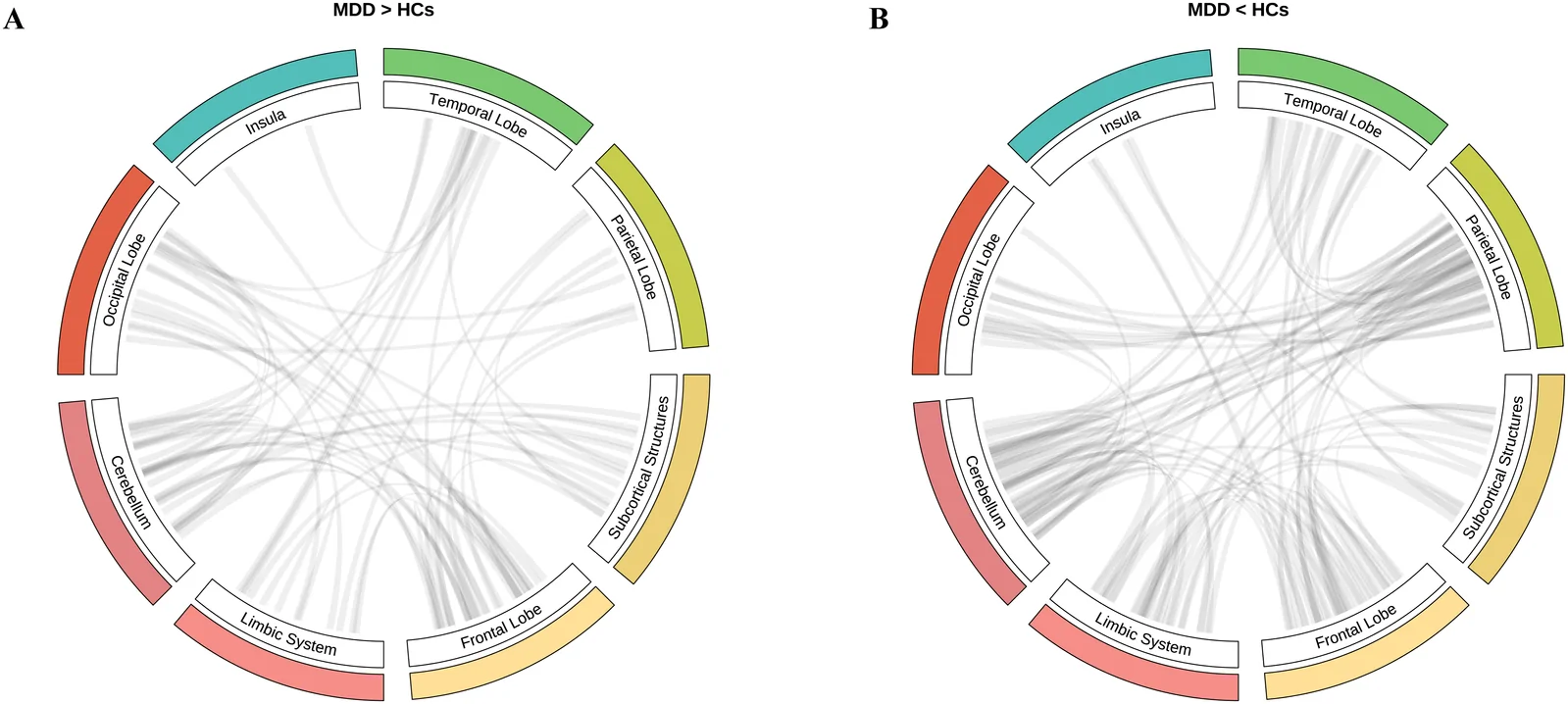
Scaffold connections changes in MDD patients; **(A)**. Significantly increased connectivity in MDD; **(B)**. Significantly reduced connectivity in MDD. MDD, major depressive disorders; HCs, healthy controls.

**Table 4:** Abnormal scaffold connections.

Region 1	Region 2	Cohens’d	p-value
MDD>HCs			
Temporal_Pole_Sup_R	Vermis_10	0.41	<0.001
ParaHippocampal_L	Pallidum_R	0.34	0.003
Frontal_Sup_L	Precuneus_L	0.34	0.003
Cingulum_Ant_L	Temporal_Mid_R	0.33	0.004
Hippocampus_R	Calcarine_L	0.33	0.004
Frontal_Inf_Oper_L	Vermis_10	0.30	0.009
Frontal_Mid_L	Precuneus_L	0.30	0.010
Frontal_Med_Orb_L	Temporal_Inf_R	0.30	0.011
MDD<HCs			
Parietal_Sup_R	Cerebelum_8_L	-0.36	0.002
Postcentral_L	Parietal_Sup_L	-0.32	0.006
Temporal_Pole_Mid_R	Cerebelum_8_R	-0.31	0.007
Frontal_Mid_R	Putamen_R	-0.31	0.007
Thalamus_L	Thalamus_R	-0.30	0.008
Cingulum_Mid_R	Caudate_R	-0.30	0.009
Precuneus_L	Temporal_Pole_Mid_R	-0.30	0.009
CereMlum_3_R	Cerebelum_10_R	-0.30	0.010

#### Classification performance

Based solely on structural network topological metrics, the SVM model achieved a mean outer-test accuracy of 66.9% [0.61–0.76], a sensitivity of 70.1% [0.37–0.92], a specificity of 63.7% [0.43–0.91], and an AUC of 74.5% [0.67–0.84] in distinguishing MDD patients from healthy controls. When using higher-order functional topology as features, the classification accuracy between MDD patients and healthy controls was 75.9% [0.68–0.86], with a sensitivity of 78.1% [0.56–0.91], a specificity of 73.9% [0.57–0.90] and an AUC of 84.9% [0.77–0.93]. The SVM model with the conventional pairwise FC achieved a classification accuracy of 64.7% [0.55–0.72], a sensitivity of 76.6% [0.44–0.95], a specificity of 53.1% [0.29–0.77] and an AUC of 70.1% [0.58–0.78]. Compared with diagnostic approaches based on topological features of brain networks from a single sequence, the classification model incorporating topological features of structural and higher-order functional brain networks achieves significantly better performance. The model fusing features achieves an accuracy of 78.9% [0.69–0.86], a sensitivity of 81.5% [0.69–0.96], a specificity of 76.4% [0.61–0.92], and an AUC of 88.2% [0.80–0.95] (**Figure 5**).

**Figure 5:**
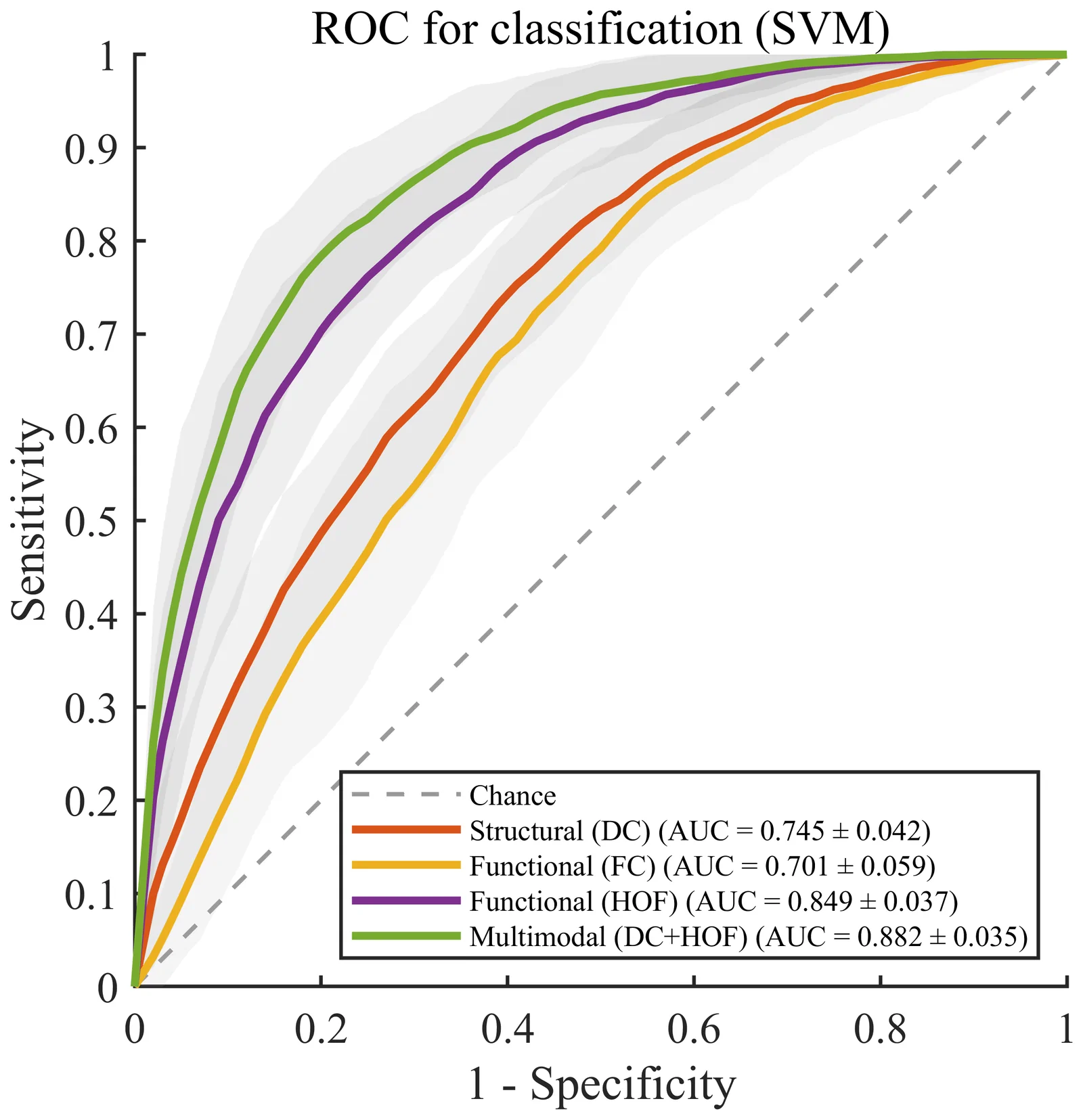
The receiver operating characteristic (ROC) curves for different models. AUC, the area under the receiver operating characteristic curve.

The DeLong test demonstrated that the multimodal model significantly outperformed the structural model (p=0.028). In addition, the HOF model achieved a significantly higher AUC than the conventional pairwise FC model (p=0.047) (**Table 5**).

**Table 5:** DeLong test for evaluating the differences between ROC curves.

Comparison	AUC 1	AUC 2	Z value	p-FDR
FC vs HOF	0.701	0.849	-2.267	0.047
DC vs DC+HOF	0.745	0.882	-2.593	0.028

#### Prediction model for MDD suicide risk

Ridge regression prediction model based on fused multimodal topological features was constructed to estimate individual suicide risk indexed by NGASR. Predicted scores showed strong positive correlation with real clinical NGASR measurements (r = 0.604 [0.499, 0.694], R^2^ = 0.364 [0.249, 0.482]) (**Figure 6**). The model yielded an RMSE of 2.330 and an MAE of 1.796. The observed NGASR scores ranged from 1 to 16 (mean ± SD, 8.17 ± 2.93).

**Figure 6:**
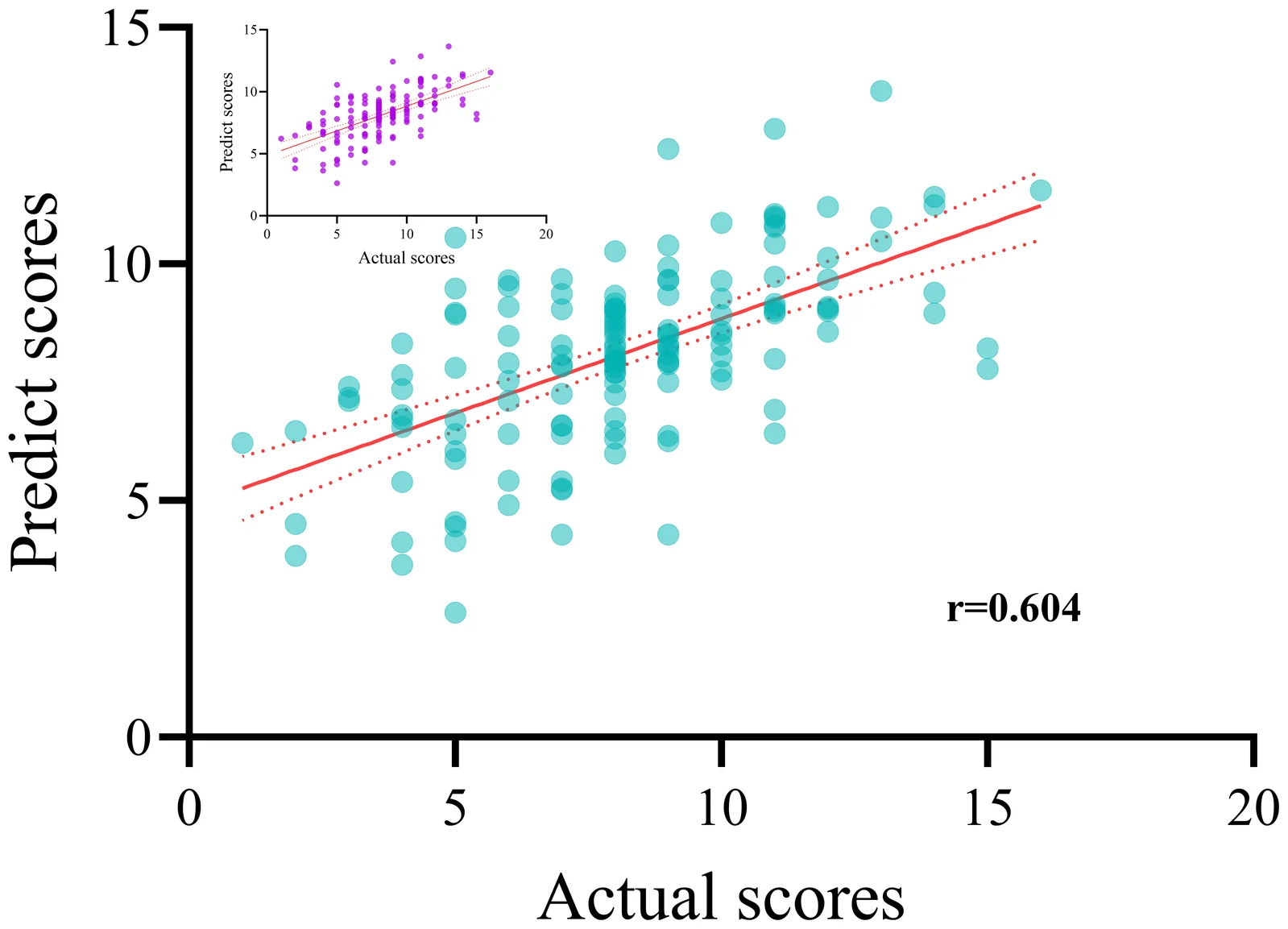
Predicting suicide risk in patients with major depressive disorder (MDD) based on neuroimaging features.

## Discussion

This multimodal MRI topological analysis integrates graph-theoretical metrics derived from DTI with persistent homology-derived higher-order functional signatures. Our results verify that the fusion of two types of topological features substantially improves MDD diagnostic power and supports quantitative prediction of suicide risk. The persistent homology pipeline adopted herein enables reconstruction of multi-node higher-order interactions from resting-state multivariate time series (32). Microscale topological signatures extracted via this method capture disease-specific brain network alterations, and the combined machine learning framework yields robust classification and predictive performance, providing new clues for unpacking the neuropathology of major depressive disorder.

At the structural network level, altered DC values were primarily observed within regions associated with the DMN, SN/VAN, and VN, as shown in the Results. DC reflects the topological importance of a node within the structural connectome and characterizes its capacity for global communication. The observed nodal alterations may indicate changes in anatomical integration across distributed brain systems involved in self-referential processing, emotional salience, sensory processing, and executive control. These findings are broadly consistent with a network-based view of depression, suggesting that MDD is associated with distributed alterations in brain organization (50, 51).

Beyond structural alterations, higher-order functional analysis revealed widespread abnormalities in bran network topology in MDD, characterized by increased violating triangles and altered homological scaffold connectivity across multiple functional networks. Violating triangles were significantly increased in the prefrontal cortex, amygdala, hippocampus, and cerebellum, suggesting altered higher-order topological organization and coordination among multiple brain regions. The DMN plays an important role in the pathophysiology of depression and is closely associated with depressive symptoms, particularly processes related to self-referential thought (52). The SN/VAN is considered important for detecting and assigning salience to emotionally relevant information and for coordinating interactions between large-scale functional networks, whereas the VN is primarily involved in visual and sensory information processing. Alterations in these networks have been repeatedly reported in MDD (53). Collectively, these findings indicate that elevated violating triangles are associated with altered coordination among distributed brain regions. Notably, the involvement of the cerebellum is a novel observation; previous studies using standard connectivity analyses have rarely implicated the cerebellum in MDD. While persistent homological scaffolds also differed between groups (as noted in Results), their functional interpretation in depression requires further investigation.

In this study, the multimodal SVM model demonstrated superior classification performance in diagnosing MDD. Integrating structural connectivity and higher-order functional topology provide complementary information by combining the anatomical basis of brain communication with higher-order network organization. Contrasted with conventional connectomic analysis, the higher-order topological measures characterize multi-region interactions, topological closure, and organizational scaffolds. These properties reflect emergent features of network organization that arise from collective interactions among multiple regions, providing additional discriminative information that complements structural network alterations.

Furthermore, our multimodal topological signatures showed reliable predictive power for NGASR-measured suicide risk in patients with MDD. Feature weighting analysis identified the frontal lobe, cerebellum, and parietal lobe as major contributors to predictive model. Our findings are broadly consistent with previous neuroimaging studies linking prefrontal and cerebellum abnormalities to suicidal ideation and suicidal behavior in MDD. In particular, alterations in prefrontal regions, including the dorsolateral prefrontal cortex, have been repeatedly reported in patients with MDD with suicidality (54). Recent evidence has also highlighted the potential involvement of the cerebellum in suicide-related and self-injurious phenotypes in MDD (55).

## Limitations

Several limitations should be acknowledged. First, the cross-sectional design precludes conclusions regarding temporal relationships or causality between neuroimaging topological alterations, depressive onset, and suicide risk. Second, although demographic covariates were controlled for, residual confounding cannot be excluded. Third, the coarse anatomical parcellation of the AAL atlas and its lack of interindividual specificity may limit the characterization of brain network topology, particularly higher-order functional networks. Future longitudinal studies are needed to validate these findings and clarify their causal mechanisms and clinical utility.

## Conclusions

In summary, integrating structural and higher-order functional topological signatures provide reliable performance for MDD discrimination and suicide risk prediction. Widespread alterations in multimodal brain topology may serve as candidate neuroimaging markers associated with MDD and suicide risk, although their specific biological and clinical significance remains to be determined. Further studies with larger, longitudinal, and independent samples are warranted to clarify the biological and clinical significance of these topological alterations and to determine their potential utility in clinical applications.

## Data Availability

The datasets presented in this article are not readily available due to data privacy concerns. Requests to access the datasets should be directed to PW, wangpeiyuan1640@163.com.
